# Chromosome-level genomes of seeded and seedless date plum based on third-generation DNA sequencing and Hi-C analysis

**DOI:** 10.48130/FR-2021-0009

**Published:** 2021-05-27

**Authors:** Weitao Mao, Guoxin Yao, Shangde Wang, Lei Zhou, Guosong Chen, Ningguang Dong, Guanglong Hu

**Affiliations:** 1 Key Laboratory of Biology and Genetic Improvement of Horticultural Crops (North China), Ministry of Agriculture, Beijing Engineering Research Center for Deciduous Fruit Trees, Beijing Academy of Forestry and Pomology Sciences, Beijing 100093, China; 2 Hubei Key Laboratory of Quality Control of Characteristic Fruits and Vegetables, College of Life Science and Technology, Hubei Engineering University, Xiaogan 432000, China; 3 School of Life Science, Hubei University, Wuhan 430062, China; 4 Hubei Key Laboratory of Food Crop Germplasm and Genetic Improvement, Food Crops Institute, Hubei Academy of Agricultural Sciences, Wuhan 430072, China; 5 Beijing XinTaoYuan Commerce & Trading Co., Ltd., Beijing 101215, China

**Keywords:** *Diospyros lotus*, genome assembly, seedlessness

## Abstract

*Diospyros lotus* L. (Date plum) is an important tree species that produces fruit with a high nutritional value. An accurate chromosomal assembly of a species facilitates research on chromosomal evolution and functional gene mapping. In this study, we assembled the first chromosome-level genomes of seeded and seedless *D. lotus* using Illumina short reads, PacBio long reads, and Hi-C technology. The assembled genomes comprising 15 chromosomes were 617.66 and 647.31 Mb in size, with a scaffold N50 of 40.72 and 42.67 Mb for the seedless and seeded *D. lotus*, respectively. A BUSCO analysis revealed that the seedless and seeded *D. lotus* genomes were 91.53% and 91.60% complete, respectively. Additionally, 20,689 (95.4%) and 22,844 (98.5%) protein-coding genes in the seedless and seeded *D. lotus* genomes were annotated, respectively. Comparisons of the chromosomes between genomes revealed inversions and translocations on chromosome 8 and inversions on chromosome 11. We identified 490 and 424 gene families that expanded in the seedless and seeded *D. lotus*, respectively. The enriched pathways among these gene families included the estrogen signaling pathway, the MAPK signaling pathway, and biosynthetic pathways for flavonoids, monoterpenoids, and glucosinolates. Moreover, we constructed the first *Diospyros* genome database (http://www.persimmongenome.cn). On the basis of our data, we developed the first high-quality annotated *D. lotus* reference genomes, which will be useful for genomic studies on persimmon and for clarifying the molecular mechanisms underlying important traits. Comparisons between the seeded and seedless *D. lotus* genomes may also elucidate the molecular basis of seedlessness.

## INTRODUCTION

Date plum (*Diospyros lotus* L.), which belongs to the genus *Diospyros* in the family Ebenaceae, is an important deciduous fruit tree species that grows in Asia, where it is cultivated for its edible fruit. The *Diospyros* genus, within the Ebenaceae (Ericales), contains more than 700 species, including the economically important persimmons (*D. kaki*, *D. virginiana*, and *D. lotus*) and ebony (*D. ebenum*)^[1−3]^. The fruit of *D. lotus* is globe shaped and yellow or bluish-black when mature^[4,5]^. Able to be grown at 2,200 m above sea level, *D. lotus* is the most cold-tolerant *Diospyros* species in China. It is used as a rootstock because of its high grafting capability and for developing new varieties because of its strong tolerance to drought and cold^[6]^. Additionally, *D. lotus* is used in drug research. The *D. lotus* fruit is used as a sedative, astringent, food and laxative, and has antiseptic, antidiabetic, antitumor, and antipyretic properties. It is also useful for treating constipation and diarrhea, dry coughs and hypertension^[7]^.

There are seeded and seedless *D. lotus* varieties, and the seedless type has a high nutritional value. The edible parts are ideal raw materials for research and the development of foods, drinks and health-care products^[8]^. Many high-quality fruits are unacceptable for consumers because they have too many seeds or their seeds are too large^[9]^. The production of seedless fruit is also attractive because it avoids the possibility of any undesirable pollination. Seedless fruit is an important and peculiar horticultural trait that has been selected for and retained during long-term cultivation. The seedless trait of fruit is very complex, and is not only affected by internal genetic factors in certain tree species and varieties, but also by external factors. Seedless fruit can be obtained using specific treatments. The addition of a certain CuSO_4_·5H_2_O concentration while cross-pollinating during the citrus flowering period significantly reduces the numbers of seeds in the fruit without affecting yield^[10]^. Similarly, watermelon fruit that results from pollination with pollen irradiated with soft-X-ray contains only empty seed, although the fruit develops to a normal size^[11]^. Additionally, gibberellic acid treatments induce parthenocarpy in Algerie loquat^[12]^.

To date, most of the research on the mechanisms underlying seedlessness has been at the cellular level, and the application of gene sequencing technology has revealed that the expression of certain genes causes fruit to be seedless. To the best of our knowledge, there are no published reports on the seedless trait of *D. lotus* fruit. Therefore, the aim of this study was to provide new insights into the production of seedless *D. lotus* fruit through genome sequencing and a comparative analysis of seeded and seedless *D. lotus* varieties. The results will also be useful for breeding *D. lotus* varieties with desirable characteristics.

## MATERIALS AND METHODS

### Plant material and DNA sequencing

**Figure 1:**
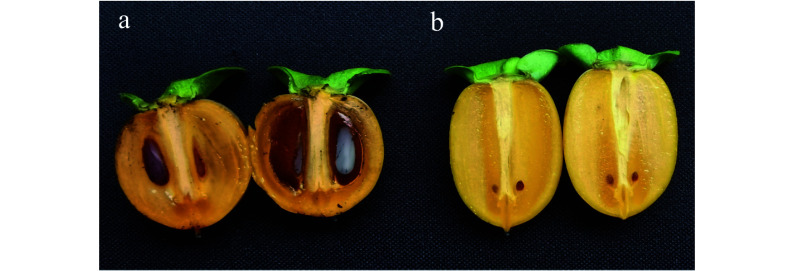
Fruit shapes and seed sizes of the seeded and seedless *Diospyros lotus*. (a) Seeded (Yz01); (b) Seedless (W01).

Two *D. lotus* varieties (Fig. 1), seedless *D. lotus* (W01) and seeded *D. lotus* (Yz01), were grown in Taoyuan Village, Zhenluoying Town, Pinggu District, Beijing, China. Fresh, healthy leaves were collected and immediately frozen in liquid nitrogen. Genomic DNA extracted from the samples using the cetyltrimethylammonium bromide method^[13]^ was used for sequencing. To obtain sufficient high-quality DNA for the PacBio Sequel II platform (Pacific Biosciences of California Inc., Menlo Park, CA, USA), the concentration and purity of the extracted DNA were determined using a NanoDrop 2000 spectrophotometer (Thermo Fisher Scientific, Waltham, MA, USA) and a Qubit fluorometer (Thermo Fisher Scientific). Moreover, the integrity of the DNA was checked by 1% agarose gel electrophoresis. The extracted DNA was sequenced on the Illumina NovaSeq 6000 (Illumina Inc., San Diego, CA, USA) and PacBio Sequel II platforms (Pacific Biosciences of California Inc., Menlo Park, CA, USA). The short reads generated from the Illumina platform were used to estimate the genome size, heterozygosity, and repeat content, whereas the long reads from the PacBio platform were used for assembling genomes. Briefly, qualified DNA samples were randomly fragmented into 350 base pair (bp) segments using ultrasonic crushing apparatus, after which they were used for the end repair, poly (A) addition, barcode indexing, purification, and PCR amplification steps. Regarding the Illumina NovaSeq sequencing analysis, we constructed a paired-end library with 150 bp sequences using the manufacturer-recommended method. After filtering, 80.99 Gb (119.53-fold genome sequence coverage) and 79.21 Gb (114.98-fold genome sequence coverage) of clean data were generated for the seedless and seeded *D. lotus*, respectively. For the PacBio sequencing, SMRTbell libraries (approximately 20 kb) were obtained according to the PacBio protocol. After removing adapters and correcting and trimming the data, 92.08 Gb (103.29-fold genome sequence coverage) and 133.51 Gb (166.98-fold genome sequence coverage) of sequence data were generated for the seedless and seeded *D. lotus*, respectively.

### RNA extraction and sequencing

Total RNA was prepared from the two *D. lotus* varieties stems, fruits and leaves using TRIzol reagent (Invitrogen, California, USA). A NanoDrop 2000 spectrophotometer (Waltham, MA, USA) and an Agilent 2100 Bioanalyzer (Agilent Technologies, USA) were applied to check RNA quality. Equal amounts of RNA from each tissue were used for cDNA library construction. Approximately 4.17 Gb and 4.15 Gb of transcript data were produced for seedless and seeded *D. lotus* from the Illumina HiSeq X Ten sequencing platform and processed using Trimmomatic (version 0.36) with the default parameters.

### Genome *de novo* assembly

Genomes were assembled using Canu (version 1.5)^[14]^, with the following parameters: maxThreads = 200, minReadLength = 1,000, corOut Coverage = 40, correctedErrorRate = 0.045, minOverlapLength = 500, rawErrorRate = 0.3, and corMin Coverage = 4. To increase the accuracy of the sequencing data, the genomes were assembled using the error correction, trimming, and assembly steps of Canu. The sequences were polished in two rounds. Specifically, the PacBio long-read sequence data were polished with Arrow^[15]^, after which the Illumina sequence data were polished using Pilon (version 1.22)^[16]^. Purge Haplotigs^[17]^ was used to remove genomic redundancies after the initial assembly and correction.

### K-mer analysis of the *D. lotus* genomes

We used Illumina short reads and a k-mer-based method^[18]^ to estimate the size, heterozygosity, and repeat content of the seedless and seeded *D. lotus* genomes, using a software package (GCE-1.0.2, https://github.com/fanagislab/GCE). The k-mer frequency (k = 17) was determined using Jellyfish software, and the frequency distribution derived from the sequencing reads was plotted.

### High-quality assembly using Hi-C technology

Fresh young *D. lotus* leaves were treated with paraformaldehyde. Chromatin was digested with the restriction enzyme *Mbo*I and ligated *in situ* after a biotinylation step. The 5′ overhangs were labeled with a biotinylated tag and repaired. Following the ligation, the DNA was extracted and sheared, after which fragments between 300 and 500 bp long were selected. The biotin-containing fragments were captured to construct a library, which was then sequenced with the Illumina system. The Hi-C library sequencing for the seedless and seeded *D. lotus* resulted in 86.96 Gb and 107.87 Gb data, respectively (Table 1). The two groups of sequencing reads were aligned to the previously assembled genomes using Bowtie2^[19]^. The Hi-C data were identified and aligned, and the repeated reads were removed. The data were filtered and evaluated in tandem using HiCUP^[20]^. On the basis of cis interactions, rather than trans interactions, contigs or scaffolds were divided, anchored, sequenced, directed and incorporated to obtain chromosome-level genomes using 3D-DNA^[21]^.

**Table 1:** Summary of the sequencing data used for assembling the *Diospyros lotus* genomes.

Library type	Seedless *Diospyros lotus* (W01)				Seeded *Diospyros lotus* (Yz01)		
	Library size (bp)	Clean data (Gb)	Coverage (×)		Library size (bp)	Clean data (Gb)	Coverage (×)
Illumina	350	80.99	119.53		350	79.21	114.98
Pacbio	20,000	92.1	103.29		20,000	133.51	166.98
Hi-C	350	86.96	−		350	107.87	−

### Genome assembly quality evaluation

To evaluate the quality of the assembled genomes, the Illumina short reads were mapped to the genomes using the BWA software^[22]^ and the PacBio long reads were mapped using BLASR^[23]^. The completeness of the assembled genomes was determined by BUSCO analyses^[24]^ using the actinopterygii_odb9 dataset. Long terminal repeat (LTR) sequences were used to evaluate genomic integrity, which was expressed as the LTR assembly index (LAI), using the LTR_finder and LTR_retriever programs^[25]^. Illumina short reads were aligned to the genome using SAMtools^[26]^, whereas Picard tools^[27]^ were used to detect mutations and GATK^[28]^ was used to count the homozygous and heterozygous SNPs and InDels. The results are herein presented as circular genomic maps.

### Genome annotation

Repetitive sequences, including transposable elements (TEs) and tandem repeats, were analyzed. More specifically, the repeated sequences in the *D. lotus* genomes were annotated using homology-based and *ab initio* prediction methods. RepeatMasker and Repeat Protein Mask (version 4.0.5)^[29]^ were used to retrieve data from the RepBase database (http://www.girinst.org/repbase). Tandem Repeats Finder^[30]^ and LTR_finder were used to make *ab initio* predictions.

The protein-coding genes were annotated using a combination of homology-based, *ab initio*, and transcriptome-based predictions. Augustus (version 3.0.2)^[31]^ was used to predict *ab initio* coding genes. For the homology-based method, protein sequences from related plants, including *Olea europaea*, *Capsicum annuum*, *Daucus carota*, *Solanum pennellii*, *Arabidopsis thaliana*, *Lactuca sativa*, and *Solanum tuberosum*, were downloaded from public databases and aligned against the *D. lotus* genomes using TBLASTN (E-value < 1e-5)^[32]^. The sequences derived from RNA-seq data were compared with the assembled *D. lotus* genomes to identify potential exon regions using TopHat (version 2.0.8)^[33]^ and Cufflinks (version 2.1.1)^[34]^. We integrated all predicted genes using MAKER software^[35]^. The following databases were screened for homologous sequences: NCBI non-redundant protein (NR), Gene Ontology (GO), Kyoto Encyclopedia of Genes and Genomes (KEGG), Eukaryotic Orthologous Groups (KOG), SwissProt, TrEMBL, InterProScan, and Pfam.

The default parameters of tRNAscan-SE^[36]^ were used to predict transfer RNA (tRNA) genes. Because ribosomal RNAs (rRNAs) are highly conserved, the rRNA sequences of related species were selected as reference sequences and used to search for rRNA sequences in the genomes via a BLASTN alignment (E-value < 1e-5). The microRNA (miRNA) and small nuclear RNA (snRNA) fragments were identified by searching the Rfam database (version 11.0)^[37]^ using INFERNAL (version 1.1)^[38]^.

### Synteny analysis of the seedless and seeded *D. lotus* genomes

The evolution of the seedless and seeded *D. lotus* chromosomes as well as gene synteny were investigated using MCScan^[39]^. A total of 17,162 gene pairs were detected in the comparisons of the seedless and seeded *D. lotus* genomes. The aligned syntenic chromosomes were visualized.

### Analysis of genome evolution

To more thoroughly examine the phylogenetic relationships of *D. lotus* and the evolution of its gene families, we clustered gene sequences from 17 related plant species and performed a phylogenetic analysis based on the protein-coding genes from the seedless and seeded *D. lotus* and the 17 other species. We extracted and downloaded the protein sequences encoded by single-copy genes from the NCBI database for the following 17 species: *Malus domestica*, *Citrus reticulata, Juglans regia*, *Solanum lycopersicum*, *Diospyros oleifera* Cheng, *Rhododendron delavayi*, *Camellia sinensis*, *Coffea canephora*, *Daucus carota*, *Coriandrum sativum*, *Cucurbita pepo*, *Vitis vinifera*, *Eriobotrya japonica*, *Sorghum bicolor*, *Arabidopsis thaliana*, *Oryza sativa* subsp. *japonica*, and *Beta vulgaris*. Analyses were conducted using BLASTP (E-value < 1e-5)^[40]^ and OrthoFinder (version 2.27)^[41]^, with an inflation parameter of 1.5. To reveal the phylogenetic relationships among *D. lotus* and the other species, the protein sequences encoded by single-copy orthologous genes were aligned using MUSCLE (version 3.8.31)^[42]^. These phylogenetic analyses were performed according to the maximum-likelihood method of PhyML (version 3.0)^[43]^. Using the molecular clock data from the TimeTree database, the divergence times were determined with the approximate likelihood calculation method of PAML (version 4.8)^[44]^. We compared the cluster size differences between the ancestors and each species and analyzed the expansion and contraction of the gene families using CAFE (version 2.1)^[45]^.

### Construction of the *Diospyros* genome database

The *Diospyros* Genome Database was set up using Tomcat and MySQL. The backend was designed and implemented using the SpringBoot + MyBatis framework, with CentOS as the server. Data were visualized using an open source ECharts package. We collected genomic data for *Diospyros oleifera* Cheng, *Diospyros lotus*_ Kunsenshi-Male, seedless *Diospyros lotus* and seeded *Diospyros lotus*.

### Data availability

The sequencing datasets and genome assemblies have been deposited in public repositories. The Illumina genome sequencing data were deposited in the NCBI Sequence Read Archive under the accession numbers SRR12450967 (Seedless) and SRR12450964 (Seeded). The PacBio genome sequencing data were deposited in the NCBI Sequence Read Archive under the accession numbers SRR12450966 (Seedless) and SRR12450963 (Seeded). The Hi-C sequencing data were deposited in the NCBI Sequence Read Archive under the accession numbers SRR12450965 (Seedless) and SRR12450962 (Seeded). The final chromosome assemblies were deposited in the NCBI GenBank database under accession numbers JACNMG000000000 (Seedless) and JACRTX000000000 (Seeded). Raw sequencing data for RNA-Seq used for annotation have been deposited in the NCBI under the SRA accession number SRR14028490 (Seeded) and SRR14026913 (Seedless).

## RESULTS AND DISCUSSION

### Genome sequencing and assembly

**Figure 2:**
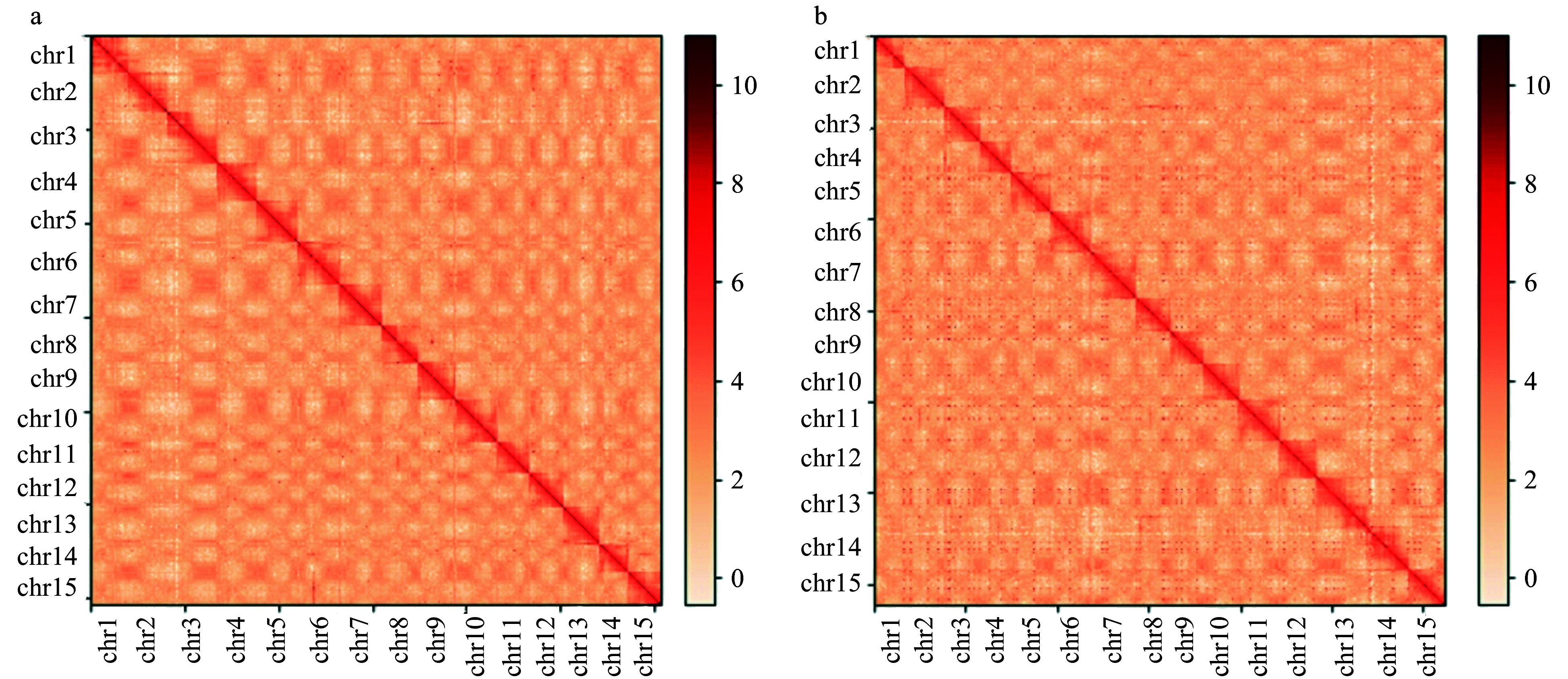
Hi-C interaction heat maps for *Diospyros lotus* genomes presenting the interactions among 15 chromosomes. (a) Seeded (Yz01); (b) Seedless (W01).

On the basis of the k-mer analysis (k-mer = 17), the seedless *D. lotus* genome size was 682 Mb, with a heterozygosity of 1.0% and a repeat content of 57.15%, whereas the seeded *D. lotus* genome size was 616 Mb, with a heterozygosity of 1.26% and a repeat content of 54.92%. A total of 92.08 Gb (103.29-fold genome sequence coverage) and 133.51 Gb (166.98-fold genome sequence coverage) of PacBio long reads, as well as 80.99 Gb (119.53-fold genome sequence coverage) and 79.21 Gb (114.98-fold genome sequence coverage) of Illumina clean data, were generated for the seedless and seeded *D. lotus*, respectively (Table 1). The total length of the assembled reads for the seedless *D. lotus* genome was 617.66 Mb, which included 706 contigs. The contig N50 was 3.01 Mb and the longest contig was 16.26 Mb. The total length of the assembled reads for the seeded *D. lotus* genome was 647.31 Mb, which included 743 contigs. The contig N50 was 2.46 Mb and the longest contig was 14.82 Mb (Table 2). The size difference between the final genomes and the genome survey sequences may have been because of the heterozygosity and repetitive sequence of the *D. lotus* genomes. On the basis of the Hi-C assisted assembly, 142 contigs were successfully clustered into 15 chromosomes in the seedless *D. lotus* genome, and the scaffold N50 reached 40.72 Mb (Supplemental Table S1), whereas 41 contigs were successfully clustered into 15 chromosomes in the seeded *D. lotus* genome, and the scaffold N50 reached 42.67 Mb (Supplemental Table S2). To the best of our knowledge, this is the first report of chromosome-level *D. lotus* genomes (Fig. 2).

**Table 2:** Summary of the assembled seedless and seeded *Diospyros lotus* genomes.

Parameter	Seedless *Diospyros lotus* (W01)			Seeded *Diospyros lotus* (Yz01)	
	Contig length (bp)	Contig number		Contig length (bp)	Contig number
N90	561,232	228		537,928	279
N80	1,144,354	151		1,078,450	194
N70	1,625,012	106		1,450,541	143
N60	2,258,638	73		2,059,392	106
N50	3,006,748	49		2,463,960	77
Total length	617,662,490	−		647,313,630	−
Number (≥ 100 bp)	−	706		−	743
Number (≥ 2 kb)	−	691		−	734
Max length	16,262,241	−		14,842,567	−

### Genome assembly quality evaluation

**Figure 3:**
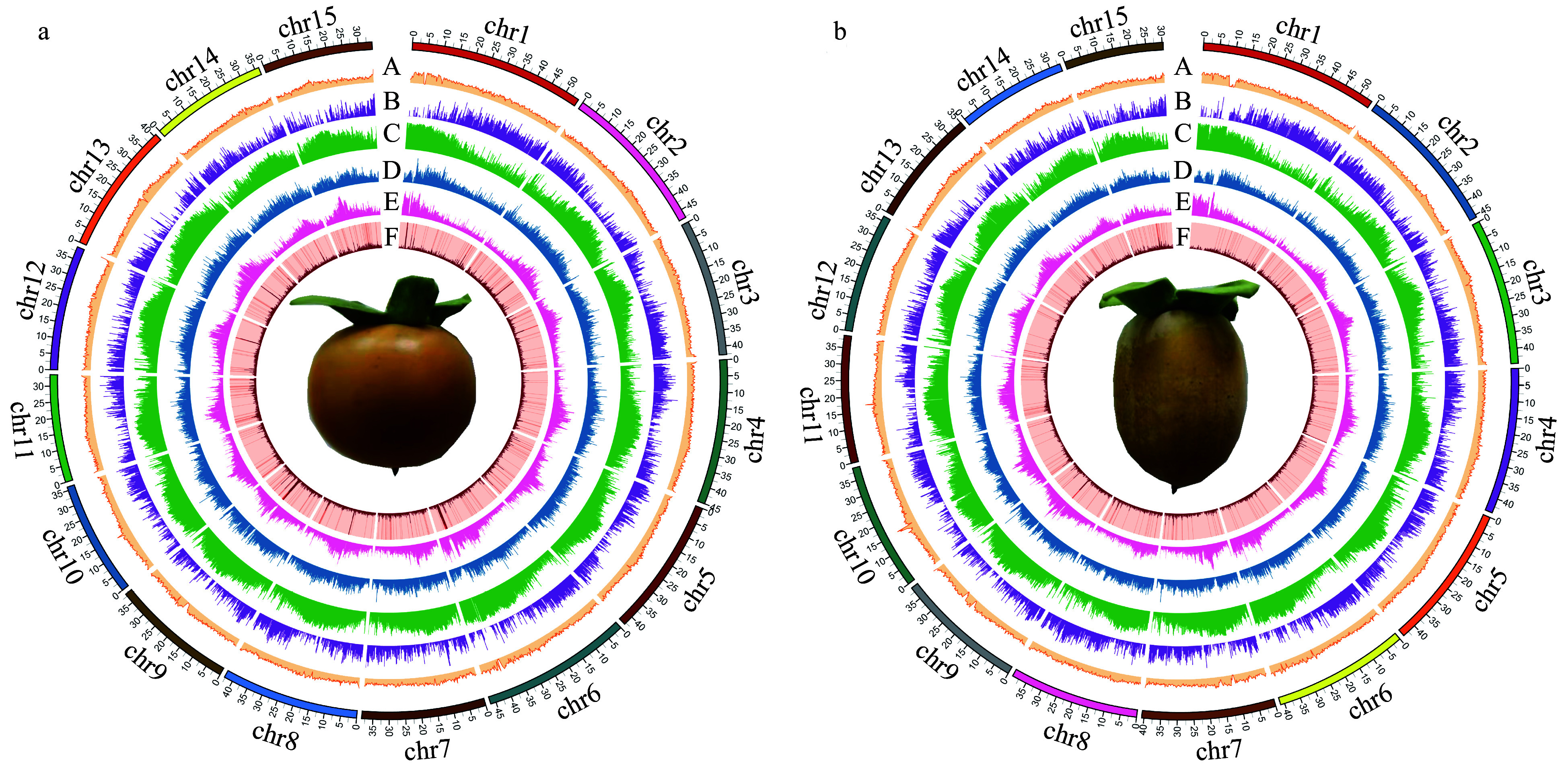
Circular genomic maps for *Diospyros lotus*. (a) Seeded (Yz01); (b) Seedless (W01). A. GC content distribution; B. Gene density distribution; C. Repeats density distribution; D. LTR-Copia density distribution; E. LTR-Gypsy density distribution; F. DNA transposon density distribution.

The Illumina short-read mapping rates were 97.74% and 98.24% for the seedless and seeded *D. lotus* genomes, respectively (Supplemental Table S3). The PacBio long-read mapping rates were 90.90% and 94.89% for the seedless and seeded *D. lotus* genomes, respectively (Supplemental Table S4). The BUSCO analysis revealed that the seedless and seeded *D. lotus* genomes were 91.53% and 91.60% complete, respectively (Supplemental Table S5). The results of the analysis of the homozygosity and heterozygosity of the SNPs and InDels are presented in Supplemental Table S4. The LAI is a newly developed reference-free genome metric for evaluating genome assembly continuity using LTR retrotransposons. The LAI values for our assembled seedless *D. lotus* genome (LAI = 15.22) and seeded *D. lotus* genome (LAI = 11.98) were relatively high, exceeding the threshold for reference genome assemblies. Circular maps of the seedless and seeded *D. lotus* genomes are presented in Fig. 3.

### Repetitive sequence annotation

A genomic analysis revealed that 69.56% of the seedless *D. lotus* genome consisted of repetitive sequences, of which TEs accounted for 68.76%. The most frequently detected TEs in the seedless *D. lotus* genome were LTR retrotransposons (55.17%), followed by DNA TEs (10.20%) (Supplemental Table S7). In contrast, 73.81% of the seeded *D. lotus* genome consisted of repetitive sequences, of which TEs accounted for 72.87%. The most frequently detected TEs in the seeded *D. lotus* genome were LTR retrotransposons (59.37%), followed by DNA TEs (11.57%) (Supplemental Table S8).

### Gene predictions and functional annotations

Homology-based, transcriptome-based, and *ab initio* gene predictions were used to generate gene models, which were combined. After eliminating sequence redundancies with MAKER, 21,684 and 23,193 protein-coding genes wereidentified in the seedless and seeded *D. lotus* genomes, respectively.The screening of the NR, GO, KEGG, KOG, SwissProt, TrEMBL, InterProScan, and Pfam databases for homologous sequences indicated that the seedless and seeded *D. lotus* genomes respectively contained 20,689 (95.41%) and 22,844 (98.50%) protein-coding genes listed in at least one public database (Table 3). The number of genes, gene length distribution, coding sequence length distribution, exon length distribution, and intron length distribution for the *D. lotus* genomes were similar to the corresponding data for the other analyzed species (Supplemental Fig. S1, S2).

**Table 3:** General statistics for the functional annotations of the genes in the seedless and seeded *Diospyros lotus* genomes.

Type		Seedless *Diospyros lotus* (W01)			Seeded *Diospyros lotus* (Yz01)	
		Number	Percent (%)		Number	Percent (%)
Total		21,684	−		23,193	−
Annotated		20,689	95.41		22,844	98.5
	InterPro	17,473	80.58		20,037	86.39
	GO	12,161	56.08		14,066	60.65
	KEGG ALL	20,547	94.76		22,750	98.09
	KEGG KO	8,435	38.90		9,812	42.31
	Swissprot	15,057	69.44		16,896	72.85
	TrEMBL	20,587	94.94		22,790	98.26
	TF	1,560	7.19		1,572	6.78
	Pfam	17,064	78.69		19,709	84.98
	NR	20,607	95.03		22,794	98.28
	KOG	17,990	82.96		20,208	87.13
Unannotated	−	995	4.59		349	1.50

### Noncoding RNA annotation

We identified snRNA, miRNA, and rRNA genes in the seedless and seeded *D. lotus* genomes based on a BLASTN search of the Rfam database (E-value < 1e-5), whereas we used tRNAscan-SE and RNAmmer to predict the tRNAs and rRNAs. Finally, 146 miRNAs, 496 tRNAs, 719 rRNAs, and 792 snRNAs were identified in seedless, with average lengths of 129, 75, 220, and 111 bp, respectively (Supplemental Table S9). Additionally, 219 miRNAs, 826 tRNAs, 2,386 rRNAs, and 1,371 snRNAs were identified in seeded, with average lengths of 127, 75, 376, and 110 bp, respectively (Supplemental Table S10).

### Synteny analysis

**Figure 4:**
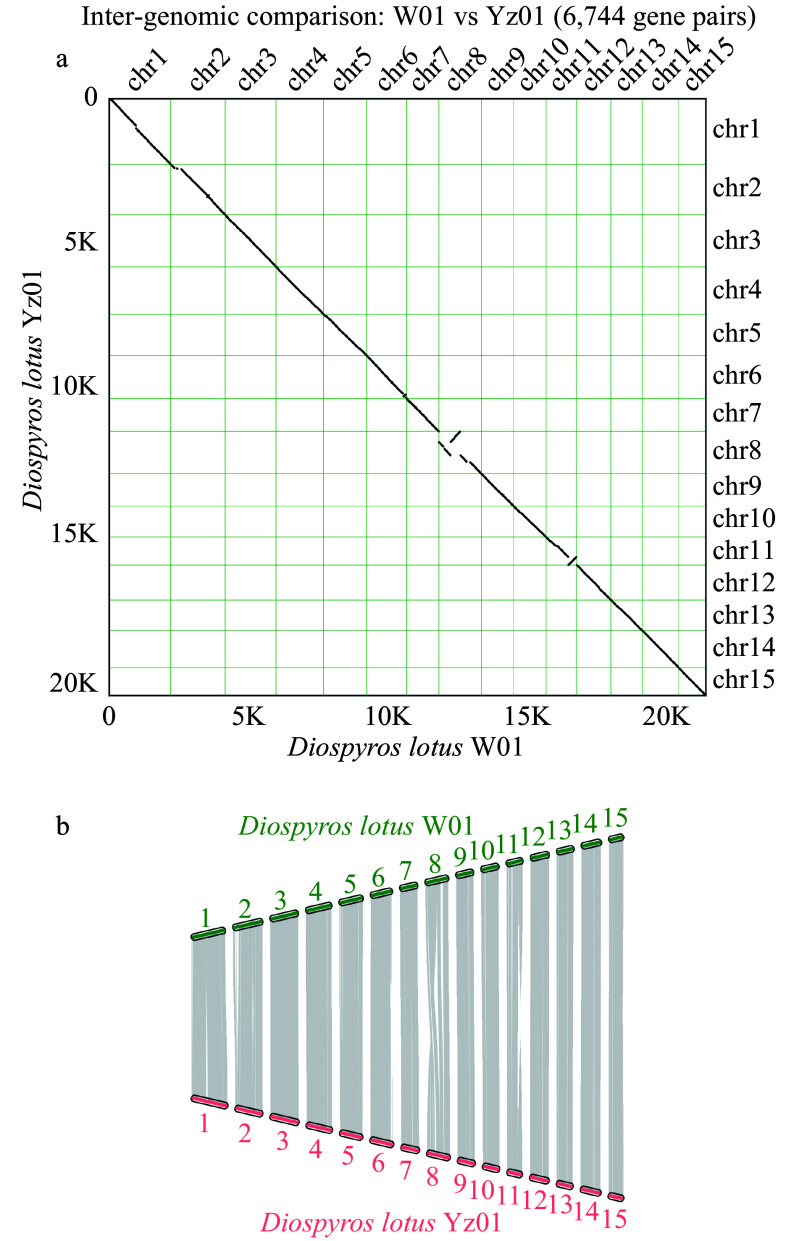
Chromosomal synteny between seedless and seeded *Diospyros lotus*. (a) Inter-genomic comparison. (b) Chromosomal maps of seedless and seeded *Diospyros lotus.*

We analyzed the synteny between the seedless and seeded *D. lotus* genomes, and the results are presented in Fig. 4b. Although the degree of synteny between the two genomes was relatively high, chromosome 8 included inversions and translocations and chromosome 11 contained inversions (Fig. 4a). These chromosomal variations may be related to differences in seedless traits, as has been reported for banana and citrus species^[9,46]^.

### Evolution of the seedless and seeded *D. lotus* genomes

**Figure 5:**
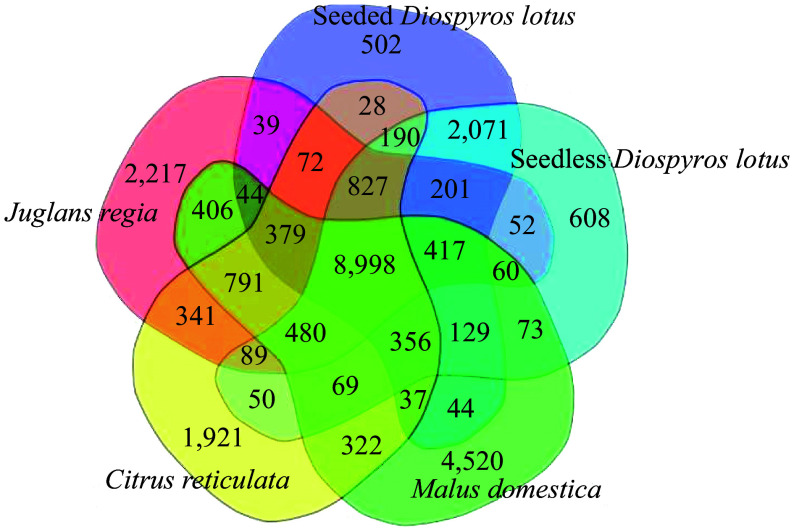
Venn diagram presenting the number of shared and unique protein-coding genes among seedless and seeded *Diospyros lotus*, *Malus domestica*, *Citrus reticulata*, and *Juglans regia* revealed by an orthology analysis.

**Figure 6:**
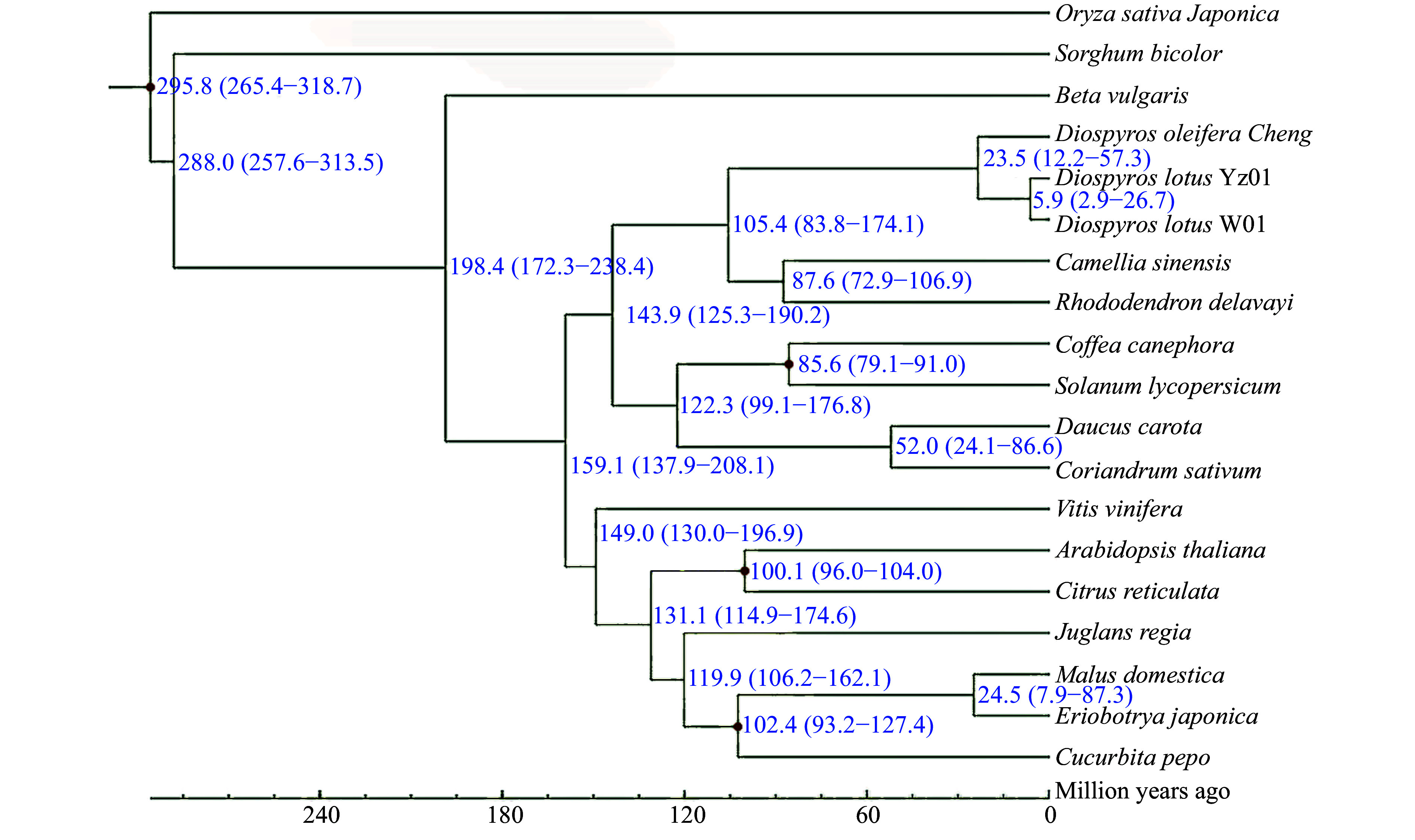
Phylogenetic tree of seedless and seeded *Diospyros lotus* and 17 other species constructed using the maximum-likelihood method. Estimated species divergence times (million years ago) and 95% confidence intervals are labeled at each branch site. Blue numbers on branches indicate the estimated divergence times. Red dots indicate the divergence times estimated based on fossil evidence.

We selected genome sequences of representative plant species for a comparative genomic analysis of seedless and seeded *D. lotus* to reveal the genome evolution and divergence time of *D. lotus.* Seedless and seeded *D. lotus* and other 17 species were analysed together (Supplemental Table S11). A total of 8,998 gene families were shared by these five species, whereas 608 and 502 gene families were unique to seedless and seeded *D. lotus*, respectively (Fig. 5). There were significantly more unique gene families in seedless *D. lotus* than in seeded *D. lotus.* The phylogenetic analysis indicated that *D. lotus* is most closely related to *D. oleifera*, with an estimated divergence time of 23.5 million years. Seedless and seeded *D. lotus* were estimated to have diverged 5.9 million years ago (Fig. 6).

### Expansion and contraction of gene families

The expansion and contraction of gene families areimportant processes during evolution^[47]^. Our analysis indicated that 490 gene families expanded in seedless (Supplemental Fig. S3). The enriched KEGG pathways among these families included estrogen signaling pathway, MAPK signaling pathway, antigen processing and presentation, longevity regulating pathway–multiple species, flavonoid biosynthesis, and metabolism of xenobiotics by cytochrome P450 (Supplemental Table S12). The enriched GO terms included cargo receptor activity;electron transporter, transferring electrons within the cyclic electron transport pathway ofphotosynthesis activity; photosynthetic electron transport in photosystem II; and scavenger receptor activity (Supplemental Table S13). Our results indicated that 1,497 gene families contracted in seedless. Fatty acid elongation and amino sugar and nucleotide sugar metabolism were two of the enriched KEGG pathways among these gene families (Supplemental Table S14). The most enriched GO term was catalytic activity (Supplemental Table S15). In contrast, our analyses indicated that 424 gene families expanded in seeded. The functional annotation of these genes revealed monoterpenoid biosynthesis, glucosinolate biosynthesis and alpha-linolenic acid metabolism were among the enriched KEGG pathways (Supplemental Table S16). Moreover, the most enriched GO terms were oxidation-reduction process, ionotropic glutamate receptor activity, extracellular ligand-gated ion channel activity and glutamate receptor activity (Supplemental Table S17). Several KEGG pathways were enriched among the 1,951 gene families predicted to have contracted in seeded, including one carbon pool by folate, nitrogen metabolism and anthocyanin biosynthesis (Supplemental Table S18), whereas the enriched GO terms were catalytic activity, serine-type endopeptidase activity and copper ion binding (Supplemental Table S19).

Seedlessness may be associated with genes related to pollen and pollination, fertilization and various hormone regulators. The expanded and contracted seedless *D. lotus* gene families included those associated with fatty acid elongation and the MAPK signaling pathway, which are important for regulating plant hormones. Lipids are part of hormone precursors, whereas auxin, ethylene and abscisic acid are correlated with the MAPK signaling pathway^[48,49]^. Therefore, analyses of the genes enriched in these pathways may provide new insights into the formation of seedless fruits.

### Database construction

**Figure 7:**
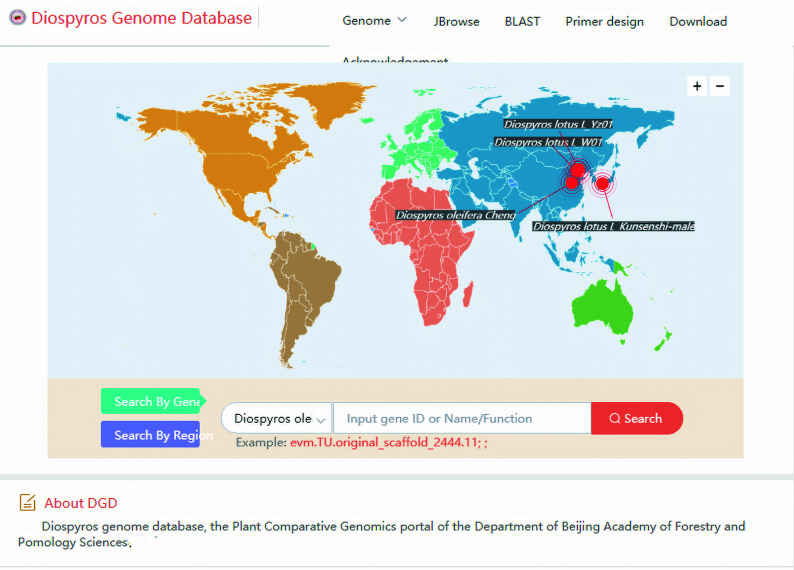
The user interface of *Diospyros* Genome Database for browsing genomes, searching for homologous sequences and designing primers.

*Diospyros*, which is the largest genus in the family Ebenaceae, comprises more than 500 economically valuable species widely distributed in the tropics and subtropics, including approximately 300 species in the Asia–Pacific region, 98 species in Madagascar and the Comoros, 94 species in mainland Africa, about 100 species in the Americas, 15 species in Australia, and 31 species in New Caledonia^[50,51]^. Many persimmon species have been studied, but relatively little research has focused on the genome. The recent increase in genome resources has produced a wealth of data for in-depth analyses of the biology and evolution of *Diospyros* plants, but obtaining and using these resources remains difficult. Thus, we developed the *Diospyros* Genome Database (http://www.persimmongenome.cn) as the first comprehensive database for *Diospyros* plant genomes. This database provides tools for browsing genomes (JBrowse), searching sequence databases (BLAST), and designing primers. To better serve the research community, wecontinue to update our database and develop new tools (Fig. 7).

## CONCLUSIONS

We applied Illumina and PacBio sequencing platforms and Hi-C technology to assemble chromosome-level reference genomes for seeded and seedless *D. lotus*. The resulting seedless and seeded *D. lotus* genomes comprised 617.66 and 647.31 Mb, respectively. The assembled seeded genome included 23,193 protein-coding genes, 219 miRNAs, 826 tRNAs, 2,386 rRNAs, 1,371 snRNAs, 424 expanded gene families, and 1,951 contracted gene families. The assembled seedless genome included 21,684 protein-coding genes, 146 miRNAs, 496 tRNAs, 719 rRNAs, 792 snRNAs, 490 expanded gene families, and 1,497 contracted gene families. We predicted that *D. lotus* and *D. oleifera* diverged approximately 23.5 million years ago, whereas seeded and seedless *D. lotus* diverged about 5.9 million years ago. The high-quality *D. lotus* genomes assembled in this study will be useful for future research on important agronomic traits among *Diospyros* species. Furthermore, comparisons between seeded and seedless *D. lotus* genomes will enable researchers to clarify the mechanisms underlying seedlessness.

## SUPPLEMENTARY DATA

Supplementary data to this article can be found online.
