# Supplementary material for: Chromosome-level genomes of seeded and seedless date plum based on third-generation DNA sequencing and Hi-C analysis
Source: For Res (Fayettev). 2021 May 27;1:9. doi: 10.48130/FR-2021-0009 (PMC11524226; doi:10.48130/FR-2021-0009)
Supplement: Supplementary file 1 — Supplementary data to this article can be found online. [file 10.48130_FR-2021-0009-Suppl-FigureS3.pdf › 10.48130_FR-2021-0009-Suppl-FigureS3.pdf]

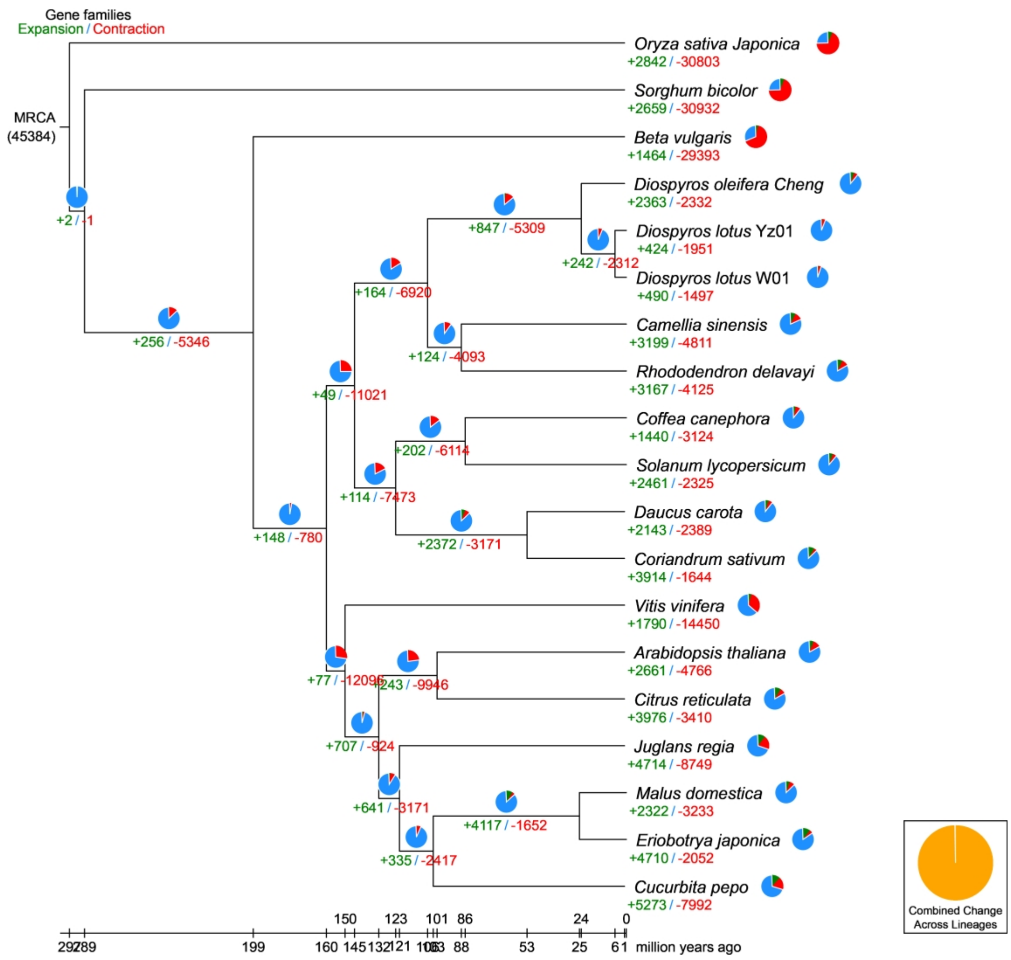

Supplementary Figure S3: Gene family expansion and contraction analysis of 19 species.Green marks the number of expanding genes, and red marks the number of contracting genes.
